# Burns in diabetic patients

**DOI:** 10.4103/0973-3930.41982

**Published:** 2008

**Authors:** Hemmat Maghsoudi, Naser Aghamohammadzadeh, Nasim Khalili

**Affiliations:** Department of Surgery, Faculty of Medicine, University of Medical Sciences of Tabriz, Iran

**Keywords:** Burn injury, diabetes, wound healing

## Abstract

**CONTEXT AND AIMS:**

Diabetic burn patients comprise a significant population in burn centers. The purpose of this study was to determine the demographic characteristics of diabetic burn patients.

**MATERIALS AND METHODS:**

Prospective data were collected on 94 diabetic burn patients between March 20, 2000 and March 20, 2006. Of 3062 burns patients, 94 (3.1%) had diabetes; these patients were compared with 2968 nondiabetic patients with burns. Statistical analysis was performed using the statistical analysis software SPSS 10.05. Differences between the two groups were evaluated using Student's *t*-test and the chi square test. *P* < 0.05 was considered as significant.

**RESULTS:**

The major mechanism of injury for the diabetic patients was scalding and flame burns, as was also the case in the nondiabetic burn patients. The diabetic burn patients were significantly older, with a lower percentage of total burn surface area (TBSA) than the nondiabetic burn population. There was significant difference between the diabetic and nondiabetic patients in terms of frequency of infection. No difference in mortality rate between diabetic and nondiabetic burn patients was observed. The most common organism in diabetic and nondiabetic burn patients was methicillin-resistant staphylococcus. Increasing %TBSA burn and the presence of inhalation injury are significantly associated with increased mortality following burn injury.

**CONCLUSIONS:**

Diabetics have a higher propensity for infection. Education for diabetic patients must include caution about potential burn mishaps and the complications that may ensue from burns.

## Introduction

Approximately 17 million (6.2%) of the US population are diabetic; this disease is the seventh leading cause of death in the US.[1] The American Diabetes Association lists the hazards associated with treating diabetic feet with hot-water bottles, heating pads, and hot water soaks but does not alert the readers to the actual severity and consequences of injuries that can ensue.[1] There have been numerous anecdotal literature reports about foot burns in diabetics due to the use of electric heating pads, foot spas, and water baths.[2–11] Diabetic patients are known to experience more infections in clean wounds than nondiabetic patients and to heal more slowly, especially in the extremities.[12–13] According to WHO, the prevalence of diabetes mellitus in the years 1995 and 2000 in Iran was 5.5 and 5.7%, respectively, and it is projected to be 6.18% in 2025. This means 1.6, 1.9, and 5.1 million affected Iranians in the mentioned years.[14] The province of East Azarbaijan is located in the northwest of Iran and covers an area of approximately 47,830 sq km. In the year 1996, this province had a population of about 3.3 million (1996 census). The mean prevalence of diabetes mellitus in our region, according to WHO criteria (1985), was 6.93%, which means that we have 228,690 diabetic patients (6930 per 100000 persons). The purpose of this study was to determine the demographic characteristics of diabetic burn patients at a large burn center (Sina Hospital) and to compare these patients with the nondiabetic burn population.

## Materials and Methods

A prospective review of all admissions to the Tabriz burn center from 20 March, 2000 to 20 March, 2006 was conducted using clinical charts. A special proforma was prepared to study the epidemiologic, demographic and therapeutic data. The patients were categorized by age, sex, %TBSA, presence or absence of inhalation injury, cause of burn, outcome, length of hospitalization, place and site of injury, need for skin grafting, date of burn, and complications.

Patients were transferred from other hospitals, brought in directly from the scene of injury, or seen earlier in the emergency room or clinic.

Resuscitative fluids were administered according to requirements, using the modified Parkland formula guidelines (for burns greater than 20% of total body surface area). All patients were treated with closed silver sulfadiazine-embedded gauze dressing (changed every 12 h) until either the wound healed or surgical intervention was initiated. Inhalation injury was determined by one or three attending surgeons based on the historical detail of each case and the clinical findings. Factors used to determine the presence of inhalation injury included burns sustained in an enclosed space, presence of facial burns, requirement for mechanical ventilation, carboxyhemoglobin level, and the presence of carbonaceous sputum. We do not use bronchoscopy and ^133^Xenon lung scan in our institution. There is no accepted way to clinically quantitate the severity of inhalation injury; the best that can be done is to determine whether inhalation injury is present or absent. We chose to use the clinical criteria that have been demonstrated to be accurate predictors of the presence of inhalation injury by Shirani *et al.* from the Brooke Army Burn Unit.[15]

During the course of hospitalization, the diagnosis of infection was based on the pathologic findings (especially, invasion of organisms into viable tissue) in wound biopsy and blood and wound culture and colony count. First, the wound was washed to remove any superficial exudates; with the use of a scalpel, a core of eschar approximately 1 cm in diameter was taken, which included obviously viable tissue of the deep margin. Care was taken to ensure that the piece had a uniform diameter so as to avoid taking an excessive amount of superficial tissue relative to deep tissue. The biopsy specimen then was wrapped in a moist sponge and immediately transported to the microbiology and pathology laboratories for processing.

Bacteremia was defined as a positive blood culture in a patient without a fever; occasionally the white blood cell count may be elevated. Sepsis was defined as a positive blood culture (that was not due to contamination) in conjunction with the clinical symptom of fever and an elevated white blood cell count. In the event of a clinical infection, empiric treatment with antibiotics was initiated against the most likely organisms until definitive culture and sensitivities were obtained. Patients with clinical signs of burn wound infection (i.e., hemorrhagic discoloration of subeschar fat, focal dark-brown or black discoloration of wound, metastatic septic lesions in unburned tissue, crusted serrations of wound margin, erythematous or violaceous edematous wound margin, conversion of second-degree burn to full-thickness necrosis) were started on antibiotics as follows: oral oxacillin (for patients who were not NPO) and parenteral cephazolin (for patients who were NPO), since the most common organism was *Staphylococcus aureus*. If the clinical sign of green pigment in subcutaneous fat was present (the clinical hallmark of *Pseudomonas aeruginosa* infection), imipenem, ciprofloxacin, and amikacin were started. Infection of diabetic burn wounds was usually caused by multiple organisms, including streptococcus, proteus, pseudomonas, methicillin-resistant *Staphylococcus aureus* (MRSA), enterococcus and enterobacter. Anaerobic cultures were ordered only if the burn wound had the distinctive odor on examination.

Diabetes was defined as (1) fasting blood glucose greater than 140 mg/dl (7.8 mmol/l) or random blood glucose levels in excess of 200 mg/dl (11.1 mmol/l) (2) For the fasting plasma glucose levels below 140 mg/l, the diagnosis established by the results of an oral glucose tolerance test, (3) with a prior (before burn injury) diagnosis of diabetes (receiving prior insulin or other therapy). Type 1 (insulin-dependent diabetes mellitus) is distinguished from type 2 diabetes (non-insulin-dependent diabetes mellitus) on the basis of the need for exogenous insulin for survival. Patients with hyperglycemia due to burn injury were excluded. All patients received insulin therapy as per their prior insulin dose (which was modified based on plasma glucose levels); In the case of those on oral hypoglycemic agents, insulin was substituted. Hyperglycemia was treated according to conventional clinical practice. This included the infusion of insulin based on a blood glucose level greater than 200 mg/dl and the maintenance of the glucose level between 70 and 110 mg/dl. Measurement of whole-blood glucose was performed daily. Additional measurements were performed if indicated. Because only 1 of 3062 patients less than 19 years of age had diabetes, the population under 19 years of age was excluded from this study.

Statistical analysis was performed using the statistical analysis software SPSS 10.05. Differences between various groups were evaluated using Student's *t*-test and the χ^2^-test. *P* < 0.05 was considered significant.

## Results

Of 3062 burn patients encountered, 94 (3.1%) were diabetic. For comparison purposes, the patients were grouped into the adult (19-50 years of age) and senior (greater than 50 years of age) groups. In the adult group, 34 of 2562 patients (1.33%) were diabetic and in the senior group, 60 of 406 patients (14.8%) were diabetic (*P* < 0.001). A total of 94 diabetic burn patients were admitted to the burn center of Sina Hospital, Tabriz, Iran from 20 March 2000 to 20 March 2006. Of the 94, 38 were males and 56 were females, with a female to male ratio of 1.47/1. The mean age was 53.4 years. There were 60 patients with type 1 DM and 34 with type 2 DM. The mean extent of burns was 17.3% ± 18.68 TBSA, with 10 patients suffering from burn sizes greater than 30% of TBSA [Tables 1 and 2]. The vast majority of injuries (46.8%) occurred at home, followed by other burns that accounted for 53.2% of admissions. Scalds resulted in 36 admissions (38.3%) and flame burns accounted for another 34 admissions (36.2%) as shown in Table 3. The main mechanisms of injury for the diabetic patients were scalding and flame burns, as is also the case in nondiabetic burn patients.

**Table 1:** Distribution of diabetic *vs* nondiabetic patients by age, frequency, mean %TBSA of burn, and mortality

Mortality %		Mean %TBSA		Percent		Frequency		Age-group (diabetic: nondiabetic)
			
Non DM	DM	Non DM	DM	Non DM	DM	Non DM	DM
24	50	31.7	55	10	4.3	298	4	19-20
28.6	25	33.3	29.5	37.9	8.5	1124	8	21-30
18.4	0	26.1	11.8	24.9	6.4	738	6	31-40
21.9	12.5	26.2	22.4	13.5	17	402	16	41-50
26.7	6.7	28.8	15.1	7.4	31.9	220	30	51-60
27.7	0	22.7	10	3.5	21.3	103	20	61-70
31.9	0	23.3	7.5	2.4	8.5	71	8	71-80
33.3	0	21.3	13	0.4	2.1	12	2	81-90
24.6	8.5	29.4	17.3	100	100	2968	94	Total

DM - Diabetic, Non DM - nondiabetic

**Table 2:** Distribution of diabetic burn patients by %TBSA, frequency, and mortality

Mortality %	Percent	Frequency	%TBSA
0	44.7	42	1-10
0	29.8	28	11-20
0	14.9	14	21-30
0	2.1	2	31-40
100	2.1	2	41-50
100	2.1	2	51-60
100	2.1	2	61-70
100	2.1	2	91-100
8.5	100	94	Total

**Table 3:** Distribution of diabetic *vs* nondiabetic patients by causes of burns, the mean %TBSA, and mortality

Mortality %		Mean %TBSA		Percent		Frequency		Causes (diabetic: nondiabetic)
			
DM	Non DM	DM	Non DM	DM	Non DM	DM	Non DM
16.7	20.3	20.2	30.4	14.9	13.9	14	413	Domestic gas
37.5	43.9	44.3	48.1	17	25.1	16	746	Kerosene
0	4.6	8.4	20.3	42.6	36.5	40	1083	Scalds
0	0	4	26	2.1	0.13	2	4	Vehicle fire
0	1.6	9.4	10.7	10.6	1.7	10	50	Hot tar
0	25.6	17	34	2.1	5.8	2	172	Benzene
0	0	4	0	2.1	0	2	0	Plant agents
0	30	27	31	2.1	2.3	2	69	Gasoline
0	2.3	17.7	10.9	6.4	3	6	88	Electrical
0	11.2	0	20.7	0	10.5	0	312	Open flam
0	0	0	6	0	0.6	0	17	Contact burns
0	14.3	0	18.9	0	0.5	0	14	Lightning
8.5	19.1	17.3	29.4	100	100	94	2968	Total

DM - Diabetic, Non DM - nondiabetic

Twenty patients had inhalation injuries and required admission to the intensive care unit for ventilatory support. The burns predominantly involved the extremities and the trunk [Table 4]. Diabetic burn patients (both adult and senior) had the same frequency of foot, upper and lower extremity, and head and torso injuries. The majority of the patients (40.42%) did not require any surgery; 20 patients (21.28%) were operated on once, 8 patients (8.5) were operated on twice, and 28 patients (29.8%) had more than two operations. The mean hospital stay was 13.04 (SD: 8.04) days; 29.8% of the patients stayed in hospital for less than 2 weeks. There was no significant difference between the diabetic and nondiabetic burn patients in length of hospital stay (13 ± 8 days *vs* 13.8 ± 13.5 days).

**Table 4:** The region of involvement in 94 diabetic burn patients

Number	Body region
4	Head
20	Face
24	Neck
18	Right arm
28	Right forearm
28	Right hand
12	Left arm
22	Left forearm
26	Left hand
20	Right thigh
22	Right leg
22	Right foot
22	Left thigh
30	Left leg
24	Left foot
26	Anterior trunk
4	Posterior trunk

There were 737 deaths, with an overall mortality of 24.1% (8 in diabetic patients *vs* 729 in nondiabetic patients). No difference in the mortality rate between diabetic and nondiabetic patients was detected when patients justified by %TBSA. The mean fatal burn size in diabetics was 68.75% TBSA, which was not significantly smaller than the mean fatal burn size (59%) in nondiabetics. Large burn size was the strongest predictor of mortality. There were 13 patients (13.8%) with inhalation injury, 6 of who died (46%). Inhalation injury was present in 6 of the 8 deaths (75%) and was significantly more common among nonsurvivors than survivors (46% *vs* 8.1%; *P* < 0.001). However, inhalation injury was strongly associated with large burns.

The diabetic burn patients were significantly older (53.4 ± 16.3 *vs* 36 ± 13.45 years; *P* < 0.001), with a lower percentage of TBSA (17.3 ± 8.1 *vs* 29.4 ± 25); *P* < 0.001) than the nondiabetic burn population. There was significant difference between the diabetic and nondiabetic patients in term of frequency of infection (14 diabetic patients (14.9%) *vs* 482 (8.1%) nondiabetic patients (*P* < 0.001). The most common organism in diabetic burns was MRSA (methicillin-resistant staphylococcus) as in the case of nondiabetic patients also [Table 5]. Diabetic patients in both age-groups were more likely to have bacteremia, sepsis, and burn wound infection. Compared with adult, senior diabetic burn patients showed a trend toward an increase in wound infections, bacteremia, and sepsis. The serum glucose level at admission was elevated in diabetic burn patients as compared with nondiabetic patients (*P* < 0.001), especially if they had an infection. Both adult and senior diabetic patients had elevated blood glucose levels with infection (147 ± 50 *vs* 282 ± 95 mg/dl) and without infection (190 ± 80 *vs* 176 ± 62 mg/dl). Nondiabetic patients also had hyperglycemia on admission (128 ± 34 mg/dl). Although the numbers were small, there was a trend for all patients with hyperglycemia (>110 mg/dl) on admission to have increased wound infections compared with patients with normal admission blood glucose levels. The elevated admission glucose values of diabetic burn patients were not predictive of the risk for later infections during the hospitalization. Wound healing is essentially normal in well-controlled diabetes but poor in patients with hyperglycemia.

**Table 5:** Comparison of organisms cultured in burn wound infections in the diabetic and nondiabetic burn population

Parameters	Burn wound infection	
	
	Diabetic	Nondiabetic
Klebsiella	2 (2.1)	22 (0.74)
Acinetobacter	0	0
Citrobacter	0	0
Streptococcus	2 (2.1)	4 (0.13)
Proteus	0	0
Pseudomonas	2 (2.1)	66 (2.2)
MRSA	0	82 (2.8)
MSSA	6 (6.4)	224 (7.5)
*E coli*	2 (2.1)	26 (0.9)
Enterococcus	0	14 (0.5)
Enterobacter	0	10 (0.34)
*H influenza*	0	0
Serratia	0	0
Staphylococcus - coagulase negative	2 (2.1)	34 (0.6)
Candida	0	0
Total	14 (14.9)	482 (8.1)

Data are *n* (%). MSSA: methicillin-sensitive *S. aureus*. Infections were clinical isolates that in conjunction with the clinical presentations originated in the following sources: wound, blood

The majority of diabetic burn patients came to the burn center during the summer and autumn; nondiabetic burn patients came throughout the year [Table 6].

**Table 6:** Distribution of diabetic burn patients by year and season

Year and Season	Frequency	Percent	Mean % TBSA	Mortality %
Year
2000-2001	6	12.8	11.2	0
2001-2002	10	21.3	25.5	20
2002-2003	24	25.5	24.6	8.3
2003-2004	13	13.8	14.4	7.7
2004-2005	10	10.6	12	10
2005-2006	31	66	15.2	6.5
Season
Spring	18	19.1	11.6	0
Summer	30	31.9	18.6	6.7
Autumn	30	31.9	18.1	13.3
Winter	16	17	11.9	12.5

## Discussion

Severe illness can cause hyperglycemia even in those patients who do not have an antecedent diagnosis of diabetes mellitus.[16] Severe stress (as during a serious illness such as severe burns) is accompanied by significant increases in the plasma concentrations of counterregulatory hormones (i.e., glucagon, epinephrine, cortisol, and growth hormone) and cytokines. These hormones cause hyperglycemia by increasing hepatic glucose production and by decreasing peripheral glucose uptake. Stress causes a greater derangement in glucose metabolism in patients with diabetes because they are not able to increase insulin secretion as a compensatory response. The exaggerated glucose response following a counterregulatory hormone infusion in healthy subjects with diabetes in comparison with nondiabetic subjects is one explanation for the deterioration in glucose control that occurs in ill patients with diabetes.[17]

There are several systemic problems that have adverse effects on tissue repair. The most recognized disease affecting healing is diabetes mellitus.[12,18] Uncontrolled diabetes results in reduced inflammation, angiogenesis, and collagen synthesis. Defects in granulocyte function, capillary ingrowth, and fibroblast proliferation have all been described in diabetes. Diabetes can affect healing by several mechanisms. First, the disease leads to both macrovascular and microvascular disease, the altered perfusion leading to impaired nutrient and oxygen delivery. Second, the peripheral neuropathy of diabetes mellitus contributes to the tendency to develop ulcers. Insensate feet will not feel minor injury, so that a minor irritation may turn into a more serious wound. Loss of the normal reflexes of the muscles that maintain the arch of the foot may lead to increased pressure areas, especially over the second metatarsal. Obesity, insulin resistance, hyperglycemia and diabetic renal failure all contribute significantly and independently to the impaired wound healing observed in diabetics. Finally, wounds in diabetic patients have a higher propensity to become infected. A minor wound frequently becomes infected and more extensive. Diabetic patients tend to have a higher risk for amputation when compared to patients without the disease. The diabetic wound appears to be lacking in sufficient growth factor levels, normal levels of which signal normal healing. It remains unclear whether decreased collagen synthesis or an increased breakdown due to an abnormally high proteolytic wound environment is responsible. Fortunately, a growth factor, PDGF-BB (Regranex™), is available to treat these wounds. The growth factor does appear to help healing in these problem wounds.[19]

In our study, diabetics were generally older and had a lower percentage of TBSA than the nondiabetic burn population. Diabetic burn patients were more likely to have cardiac and hypertensive comorbidities compared with nondiabetic burn patients. This study did not delineate whether the increased comorbidities of the diabetic burn patients contributed to longer hospitalization, which then predisposed them to infections. Elevated glucose levels were present in all diabetic burn patients on admission and were frequently difficult to control during hospitalization. As expected, with infections, some patients required more rigorous hyperglycemic control with insulin drips. This study indicated that the diabetic burn population was more susceptible to complications such as infections. Although the infections were frequently due to multiple organisms, *S aureus* was seen to be a major infective agent in burn wounds.[20] None of the studied diabetic burn patients suffered significant anaerobic infections. The burn wound was generally acute and a surface lesion; only the occasional patient presented with anaerobic or fungal infections in the burn wound since anaerobes die readily on exposure to air. There have been specific associations of diabetic patients with anaerobic cholecystitis and osteomyelitis,[21] however there were no cholecystitis and osteomyelitis cases in our study. Diabetic burn patients were more likely to develop *Clostridium difficile*-associated diarrhea, for which they were treated with metronidazole. It was not possible to determine whether there was any predilection of any organism for a particular infection site since patients often had a variety of organisms growing in different sites. This study did not evaluate any precipitating factors that may have contributed to invasion with any particular organism.

There are several reports that diabetic burn patients have a higher mortality rate than nondiabetic patients with comparable burn size. Our study found no difference.

This study indicates that increasing %TBSA involvement and the presence of inhalation injury are significantly associated with increased mortality following burn injury (in both diabetic and nondiabetic burn patients). In addition, we demonstrated that in our series of 94 diabetic burn patients that the most important predictor of mortality following thermal injury is %TBSA. The presence or absence of inhalation injury has little impact on the accuracy of this prediction. Patient gender, age, cause of burn, and the presence or absence of diabetes was not predictive of mortality.

A recent study in pediatric patients demonstrated an association of hyperglycemia with positive blood cultures, reduced graft take, and increased mortality.[22] Another study reported a significantly reduced graft take in adult burn patients with hyperglycemia.[23] The failure of growth hormone therapy as anabolic treatment in patients with burns supports these findings.[24] Growth hormone substantially aggravates insulin resistance and hyperglycemia[25] and doubles the mortality rate among critically ill patients, mainly because of organ failure and sepsis.[26] The findings of our study do not confirm these results.

A direct cause-and-effect relationship between poor glucose control and increased mortality could not be made out, which may be due to the large number of physiological variables that determine glucose metabolism. In conclusion, even though the exact mechanisms remain speculative and existing studies fail to establish cause and effect, adverse effects of diabetes and insulin resistance are found consistently.

The older diabetic patients were more likely to sustain their burns during falls in the shower or bath, with perineal, buttock, and upper thigh burns. They required urinary catheters, which predisposed them to urinary tract infections. This study confirms the findings presented in another diabetic burn study.

Careful correction of blood glucose levels improves the outcome of wounds in diabetic patients. Increasing the inspired oxygen tension, judicious use of antibiotics, and correction of other coexisting metabolic abnormalities can all result in improved burn wound healing.

In summary, diabetes in burn patients is common and may adversely affect outcomes. Further research should focus on optimal management of diabetic burn patients. It is estimated that 20% of all burn patients treated are diabetics who cannot easily distinguish hot from warm due to loss of feeling in the lower extremities. It only takes a few seconds to check the water temperature with a thermometer, but those few seconds could save a diabetic weeks or months of painful recuperation.

It is imperative that the diabetic population is educated about the hazards and complications of burn injuries. More preventive patient education addressing thermal injury, frostbite, wound healing, and infections in diabetic patients is indicated to decrease the number of accidental injuries and the resulting hospitalization.
